# Supplementary material for: An Oral FMT Capsule as Efficient as an Enema for Microbiota Reconstruction Following Disruption by Antibiotics, as Assessed in an In Vitro Human Gut Model
Source: Microorganisms. 2021 Feb 11;9(2):358. doi: 10.3390/microorganisms9020358 (PMC7918368; doi:10.3390/microorganisms9020358)
Supplement: Supplementary file 1 [file microorganisms-09-00358-s001.zip › Supplementary figure 1.pdf]

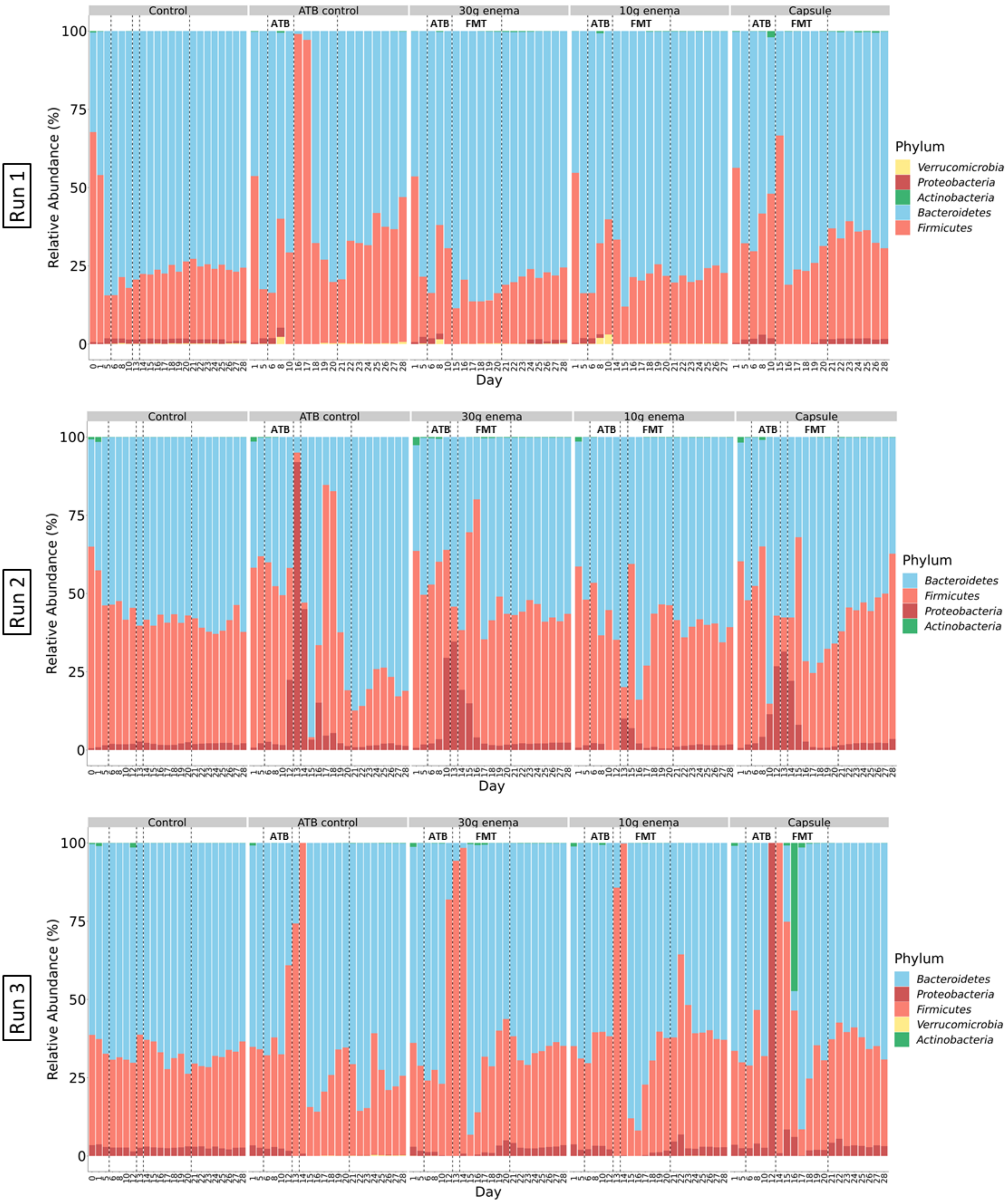

Supplementary Figure 1: Effect of FMT treatment on microbial composition at phylum level

Five bioreactors were inoculated with fecal suspension from a unique donor and ran in parallel for 28 days. The experiment was done in triplicate with fecal samples from three healthy adult

donors (donor 1, 2 and 3 in A, B and C, respectively). Different conditions were applied: no treatment (control), addition of ciprofloxacin from day 6 to day 12 (ATB control), addition of ciprofloxacin followed by FMT treatment with 30 g enema at days 14 and 15 (30 g enema), addition of ciprofloxacin followed by FMT treatment with 10 g enema at days 14 and 15 (10 g enema), and addition of ciprofloxacin followed by FMT treatment with oral capsules three times from day 14 to day 20 (capsule). Microbial composition was determined at the phylum level by 16S rRNA gene sequencing and expressed as relative abundances.
