# Supplementary material for: An Oral FMT Capsule as Efficient as an Enema for Microbiota Reconstruction Following Disruption by Antibiotics, as Assessed in an In Vitro Human Gut Model
Source: Microorganisms. 2021 Feb 11;9(2):358. doi: 10.3390/microorganisms9020358 (PMC7918368; doi:10.3390/microorganisms9020358)
Supplement: Supplementary file 1 [file microorganisms-09-00358-s001.zip › Supplementary Figure 2.pdf]

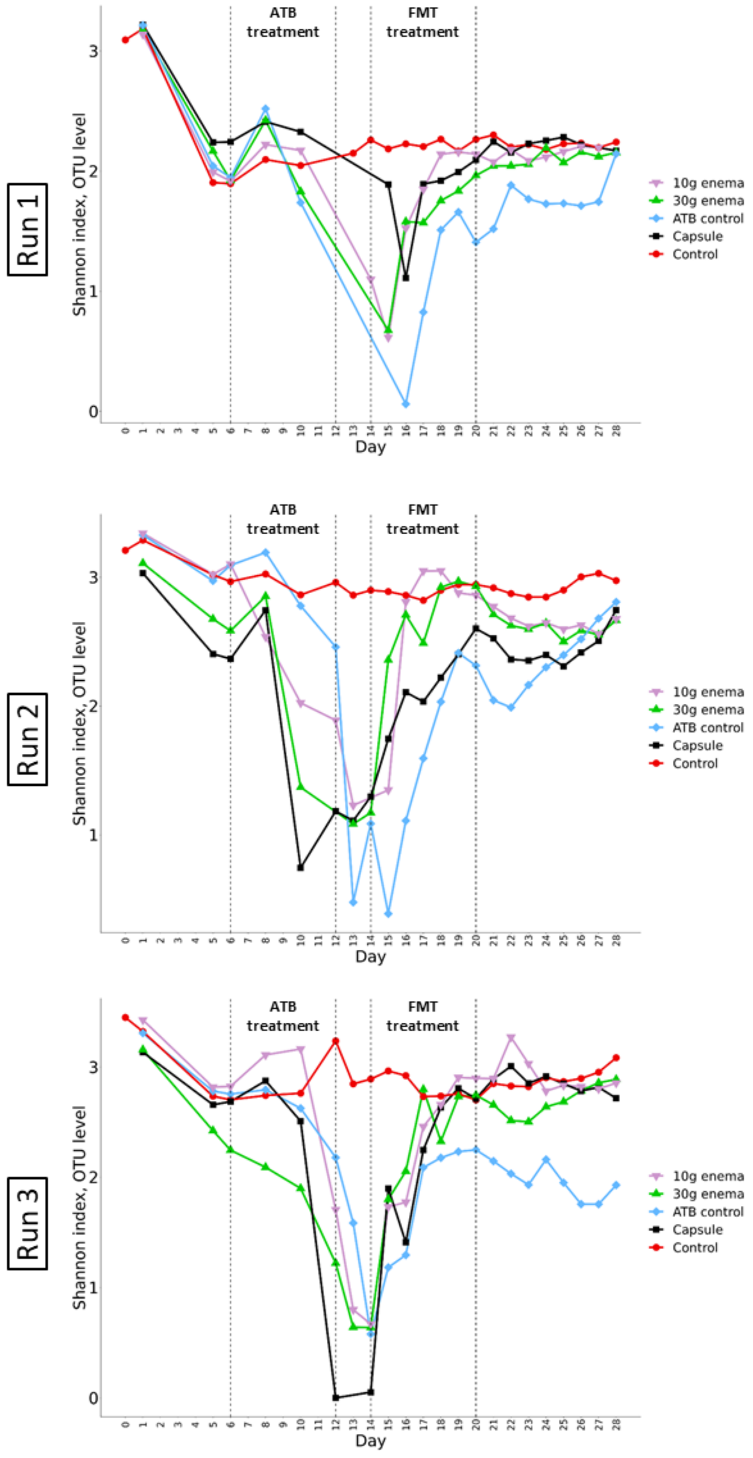

Supplementary Figure 2: Effect of FMT treatment on Shannon index at OTU level

Experiments were performed as described in Supplementary Figure 1 in triplicate with fecal samples from three healthy adult donors (Run1, Run2 and Run3). Different conditions were applied: no treatment (control, red), ciprofloxacin (ATB control, blue), ciprofloxacin and 30 g enema (green), ciprofloxacin and 10 g enema (purple), and ciprofloxacin with oral capsules (black). Shannon indexes were determined at OTU level after 16S rRNA gene sequencing.
